# Different Methods for Calculation of Activation Energies During Non-Isothermal Annealing of Mg_72_Zn_27_Pt_1_ and Mg_72_Zn_27_Cu_1_ Metallic Glasses

**DOI:** 10.3390/ma18030694

**Published:** 2025-02-05

**Authors:** Aleksandra Pierwoła, Janusz Lelito, Michał Szucki, Halina Krawiec

**Affiliations:** 1 Faculty of Foundry Engineering, AGH University of Krakow, 30 Mickiewicza Street, 30-059 Kraków, Poland; krawiec@agh.edu.pl; 2 Foundry Institute, Technische Universität Bergakademie Freiberg, 4 Bernhard-von-Cotta-Str., 09599 Freiberg, Germany; michal.szucki@gi.tu-freiberg.de

**Keywords:** metallic glasses, activation energies, Mg_72_Zn_27_Pt_1_, Mg_72_Zn_27_Cu_1_

## Abstract

Mg_72_Zn_27_Pt_1_ and Mg_72_Zn_27_Cu_1_ metallic glasses were produced using a melt-spinner. Their crystallization kinetics were investigated during annealing with five heating rates using DSC. Amorphous Mg_72_Zn_27_Pt_1_ crystallized in the form of one and Mg_72_Zn_27_Cu_1_ crystallized in the form of two exothermic crystallization peaks. It was noticed that the glass transition, the onset crystallization and the crystallization peak temperatures were strongly heating-rate-dependent. The addition of Pt and Cu increased the stability compared to that of binary Mg-Zn glass, and especially so with Pt, due to its higher melting point and different atom size to those of Mg and Zn. The activation energies were calculated using six model-free methods: the Kissinger, Ozawa–Flynn–Wall, Boswell, Tang, Augis–Bennett and Gao–Wang methods. The Augis–Bennett and Gao–Wang methods allow for the calculation of only the activation energy at the crystallization peak but they are the only ones that consider $T_{x}$ or dx/dT. For Mg_72_Zn_27_Pt_1_, the calculated values fluctuate in the ranges 114.60–117.99 kJ/mol, 102.46–105.98 kJ/mol and 71.16–98.62 kJ/mol for $E_{g}$, $E_{x}$ and $E_{p}$, respectively, whereas, for Mg_72_Zn_27_Cu_1_, the calculated values are in the ranges of 98.51–101.77 kJ/mol, 95.15–98.51 kJ/mol and 55.15–93.34 kJ/mol for $E_{g}$, $E_{x}$ and $E_{p}$, respectively. Both alloys are meta-stable in the amorphous state and crystallization occurs spontaneously. The Kissinger, Ozawa–Flynn–Wall, Tang and Boswell methods give similar values for the activation energy. The Gao–Wang method significantly underestimates values compared to other methods. The Augis–Bennett method shows much lower values for the local activation energy. Considering the ease of their formulas, best convergence and widespread use in the literature, the Kissinger and Ozawa–Flynn–Wall methods will work very well for any comparison.

## 1. Introduction

In recent years, growing interest in metallic glasses has been observed. This is due to their unique properties such as their high strength or high corrosion and abrasion resistance [1,2,3]. Amorphous metals are alloys without a long-range atomic arrangement, obtained usually by rapid solidification. However, metallic glasses are energetically meta-stable materials. Their non-crystalline state can easily transform into crystalline over time, especially with increasing temperature. This involves a complete change in their properties. The thermal stability and properties of each metallic glass strictly depend on its chemical composition [1,2,3].

The crystallization behavior and the thermal stability of amorphous alloys have still not been thoroughly investigated, although many studies have been conducted. Differential Scanning Calorimetry (DSC) is the most frequently used technique to attempt to understand these issues [1,4]. The interpretation of DSC thermograms designates values to characteristic temperatures such as the glass transition temperature $T_{g}$, onset crystallization temperature $T_{x}$, crystallization peak temperature $T_{p}$ and melting point $T_{m}$. The data from thermograms can be used to determine the kinetic parameters of the analyzed process like the activation energy, reaction model, reaction constants or pre-crystallization kinetics [1,2,4]. DSC experiments can be carried out in both isothermal and non-isothermal conditions. To calculate the activation energy of a transition, it is necessary to use annealing with a few different heating rates.

The activation energy of a transition is an important kinetic parameter during the analysis of the crystallization of the amorphous alloy. This corresponds to the value of energy that is necessary to overcome the energy barrier and initiates, e.g., the rearrangement of atoms, nucleation or crystal growth [3]. In the literature, there are several methods used to calculate the activation energy of reactions. The main classification of these methods involves isokinetic techniques (e.g., the JMA model [5,6] and the Matusita–Sakka [7]) and iso-conversional techniques (e.g., the Kissinger [8], Ozawa–Flynn–Wall [9,10], Boswell [11], Tang [12], Augis–Bennett [13], Gao–Wang [14] and Friedman methods [15]). In isokinetic (model-fitting) methods, the reaction rate is constant over a full range of temperatures or times. Thus, the kinetic parameters are assumed to also be constant with time and temperature. However, in iso-conversional (model-free) methods, the reaction rate is only a function of temperature (for a constant degree of transformation), but kinetic parameters are dependent on the transformation degree at any time and the temperature.

Numerous metallic glasses have been synthesized in binary, ternary, quaternary and more complex alloy systems, but the relative ease of the glass transition is guaranteed by the choice of more element systems [2]. So far, Mg_72_Zn_27_Pt_1_ and Mg_72_Zn_27_Ag_1_ amorphous alloys have, in part, been examined, with particular emphasis on the crystallization mechanism (using DSC and the Jeziorny–Avrami method) and the interpretation of phases arising during the heating process (using XRD) [16]. This article aims to determine the values of characteristic and local activation energies during non-isothermal annealing of amorphous Mg_72_Zn_27_Pt_1_ and Mg_72_Zn_27_Cu_1_ alloys with different heating rates. In this work, only the first Mg_72_Zn_27_Cu_1_ crystallization peak was analyzed for a representative comparison for both alloys. For the calculation, six iso-conversional methods (Kissinger, Ozawa–Flynn–Wall, Boswell, Tang, Augis–Bennett and Gao–Wang) were used and all the results were compared to each other. The aim is to explain the differences between the mentioned methods and to indicate the most advantageous one.

## 2. Materials and Methods

The crystalline base alloys were prepared by melting in an induction furnace under argon. The metal was cast in a cylindrical metal mold. Samples up to 10 mm wide were cut from the ingots. The samples were rinsed in acetone and dried, then placed in a glass capsule. The prepared capsules were placed individually in a melt-spinner with a built-in induction furnace and a copper wheel for rapid solidification. The linear speed of the wheel was 50 m/s. The thin ribbons of amorphous Mg_72_Zn_27_Pt_1_ and Mg_72_Zn_27_Cu_1_ alloy were manufactured under an argon atmosphere.

The ribbon amorphousness was confirmed using X-ray diffraction by a Panalytical Empyrean diffractometer equipped with a Cu Kα X-ray source. Additionally, amorphousness was confirmed during SEM observations using a TESCAN VEGA 3 microscope (Brno, Czech Republic).

Then, five specimens of the ribbon of about 10.2–17.1 mg weight were individually annealed from room temperature to 723 K at heating rates *β* = 5, 10, 20, 40 and 80 K/min. The thermal behavior of these samples was investigated using a differential scanning calorimeter TA DSC Q20 (Eschborn, Germany) under a flowing argon gas atmosphere (40 mL/min). All required parameters for applying the kinetic equations were recorded by DSC during the experiment.

## 3. Results

**Figure 1:**
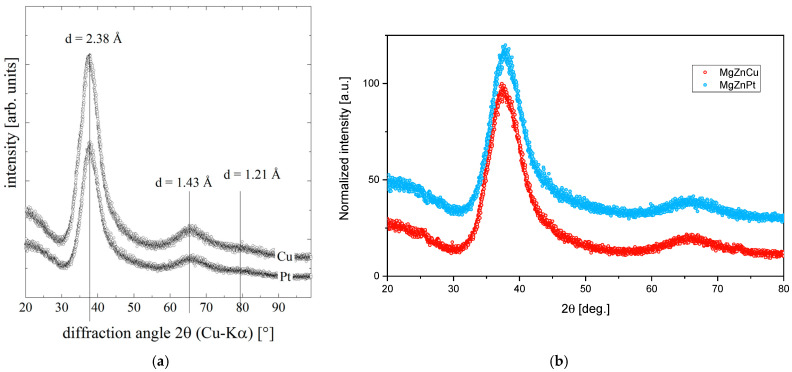
XRD patterns of amorphous Mg_72_Zn_27_Pt_1_ and Mg_72_Zn_27_Cu_1_ before (**a**) and after (**b**) normalization.

**Figure 2:**
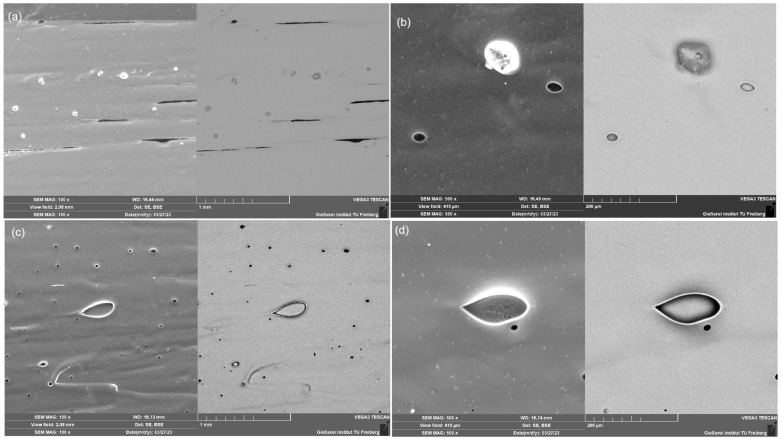
SEM images for Mg_72_Zn_27_Pt_1_ metallic glass at magnifications of (**a**) ×100 and (**b**) ×500, and Mg_72_Zn_27_Cu_1_ metallic glass at magnifications of (**c**) ×100 and (**d**) ×500.

On the XRD pattern of amorphous Mg_72_Zn_27_Pt_1_ and Mg_72_Zn_27_Cu_1_ (Figure 1a), the wide and high halo peaks are visible in the area equal to 32–45 and 60–70. There are no significant crystallinity peaks, which confirms the amorphous structure of the samples. However, there is a visible variation in the intensities for those samples originating from different measurement time; hence, it is hard to tell the difference. Therefore, normalization of the XRD patterns is helpful to judge the difference between them (Figure 1b). As is apparent, the differences are marginal, suggesting similar disorder in both alloys. Moreover, the SEM images presented in Figure 2 allow one to assume that no visible grains appeared in the structure of the produced metallic glasses.

**Figure 3:**
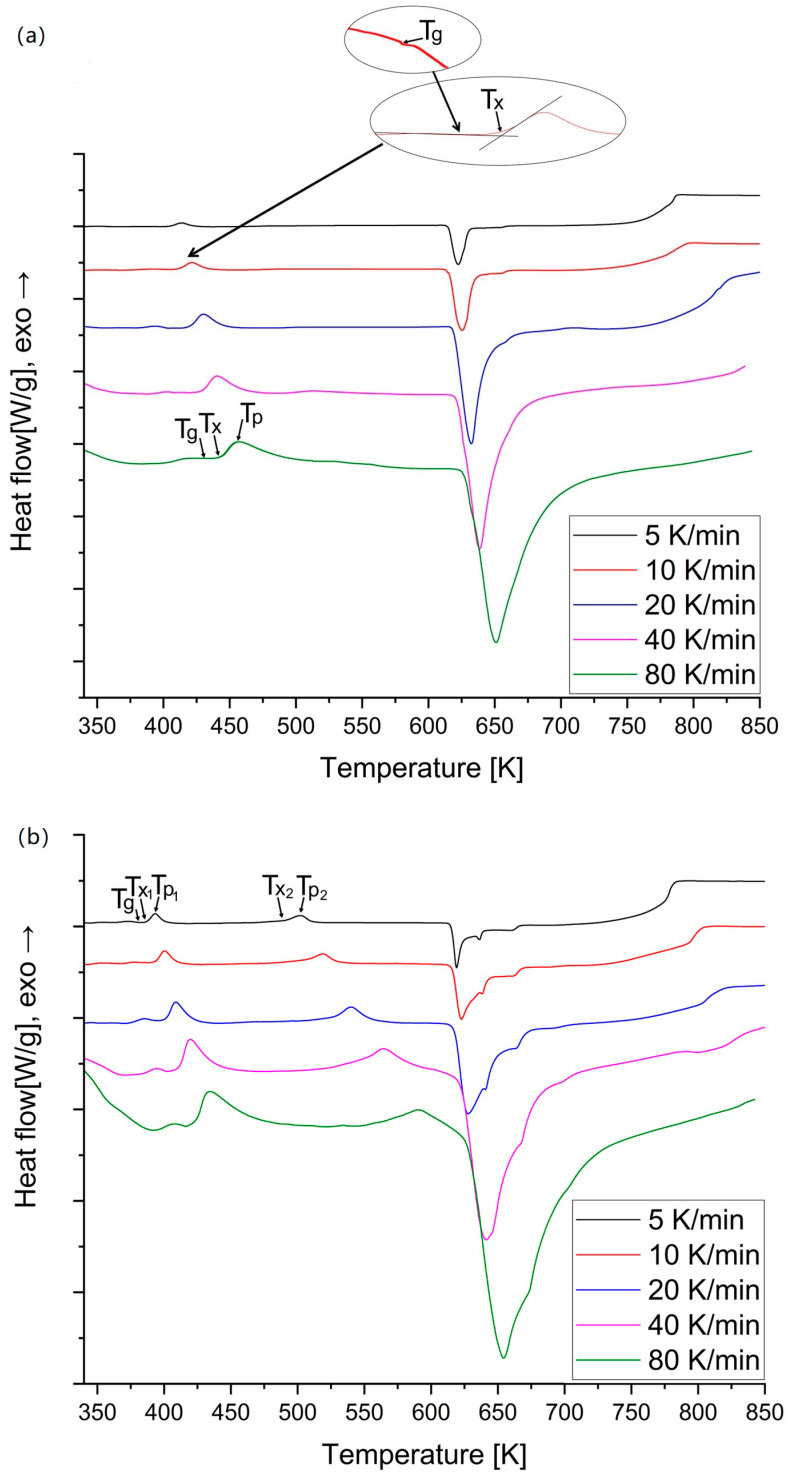
The DSC traces of amorphous (**a**) Mg_72_Zn_27_Pt_1_ and (**b**) Mg_72_Zn_27_Cu_1_ at various heating rates: 5, 10, 20, 40, 80 K/min.

The DSC thermograms of the amorphous Mg_72_Zn_27_Pt_1_ and Mg_72_Zn_27_Cu_1_ for various heating rates are shown in Figure 3. On the DSC thermogram, there is only one exothermic peak that matches the crystallization (Figure 3a). However, high-temperature XRD research on amorphous Mg_72_Zn_27_Pt_1_ [16] showed reflections corresponding to α-Mg and Mg_12_Zn_13_, whereas two exothermic peaks corresponding to crystallization and one endothermic peak corresponding to melting are visible on each curve for Mg_72_Zn_27_Cu_1_ (Figure 3b). This work will only include calculations for the first crystallization peak of Mg_72_Zn_27_Cu_1_.

The values of the glass transition temperature $T_{g}$, onset crystallization temperature $T_{x}$, crystallization peak temperature $T_{p}$ and end of crystallization temperature $T_{x\_end}$ are estimated and compiled in Table 1. β [K/min] represents the heating rate. In order to determine $T_{g}$, a bend in the curve was sought before the crystallization peak occurred. $T_{g}$ is the midpoint between heat flow baselines (inset in Figure 3a). In order to determine $T_{x}$, two tangents were determined, first at the baseline and the second along the increasing side of the peak; $T_{x}$ is their intersection (inset in Figure 3a). $T_{p}$ corresponds to the temperature at the maximum of the peak. To estimate $T_{x\_end}$, two tangents were used, similarly to $T_{x}$, first on the decreasing side of peak and the second at the baseline in the melting direction; the value sought is the intersection of the tangents. Additionally, in the curves presented in Figure 3, it can be observed that the amount of heat obtained from the endothermic peak is about three times greater than the total amount of heat obtained from the exothermic peaks. A. Calka et al. in their publication [17] observed the same phenomenon. This phenomenon is probably due to the large excess specific heat of the supercooled liquid relative to the solid and perhaps a small loss of enthalpy during heating to the crystallization temperature.

The values of $T_{g}$, $T_{x}$, $T_{p}$ and the end of crystallization temperatures $T_{x\_end}$ are estimated using tangents and are compiled in Table 1. All the mentioned temperatures increase with the heating rate. Moreover, at lower heating rates, the crystallization peaks are small and narrow, which means that at higher heating rates, a greater thermal effect is achieved. This shows that the transition behavior of amorphous Mg_72_Zn_27_Pt_1_ and Mg_72_Zn_27_Cu_1_ alloy is dependent on the heating rate. This is caused by the thermal activation of the nucleation and grain growth for the crystallization process and amorphous relaxation for the glass transition area [3].

The data given in Table 1 allow one to determine the thermal stability of the amorphous phase. The thermal stability of the amorphous phase can be estimated based on the glass transition temperature ($T_{g}$), crystallization temperature ($T_{x}$) and $∆T_{x}=T_{g}-T_{x}$, which is defined by the temperature difference between $T_{g}$ and $T_{x}$. $∆T_{x}$ is the area of the supercooled liquid before crystallization during heating of the amorphous phase. For the platinum-containing alloy, $∆T_{x}$ varies from 10.92 K to 18.77 K, while for the copper-containing alloy, $∆T_{x}$ varies from 10.56 K to 13.95 K. Since the large $∆T_{x}$ of the amorphous alloy is attributed to the slow kinetics of nucleation and growth in the supercooled liquid, the high glass forming ability (GFA) of the amorphous alloy can be inferred from the large $∆T_{x}$.

The addition of platinum and copper increases the $T_{x}$ value compared to that of binary amorphous Mg_72_Zn_28_ [18] to about 48–62 K (dependent on the heating rate) and 30–43 K for Mg_72_Zn_27_Pt_1_ and Mg_72_Zn_27_Cu_1_, respectively. The reason for this is the higher melting point for platinum (1768.3 °C/2041.4 K) [19] and zinc (1084.6 °C/1358.7 K) [19].

There have been some other DSC studies on three- and four-component amorphous alloys with bases of Mg and Zn. The addition of calcium in amorphous Mg_72_Zn_24_Ca_4_ [20] significantly shifts the onset of crystallization towards higher temperatures by ~100 K. Similarly to the investigated alloys, the characteristic temperatures increase with heating rates. Two overlapping peaks are also visible for 5 and 10 K/min, but for 20–80 K/min, there is only one crystallization peak. For three amorphous alloys with the addition of 4 at% of Ag [21], a non-isothermal experiment was performed with heating rates 5, 10, 20 and 40 K/min. The characteristic temperatures also increase with heating rates. For Mg_73_Zn_23_Ag_4_ and Mg_70_Zn_26_Ag_4_, there are also two overlapping peaks visible, but, for Mg_67_Zn_28_Ag_4_, there exists a third peak in the range 481.1–501.2 K. The relative current volume of crystalline phase *x* was calculated using definite integrals, as follows [22]:

(1) $$x=\frac{\int_{T_{x}}^{T}\frac{dH}{dT}dT}{\int_{T_{x}}^{T_{x\_end}}\frac{dH}{dT}dT}$$

where T [K] is a given temperature, $T_{x}$ [K] is the onset of crystallization, $T_{x\_end}$ [K] is the end of crystallization and H [W/g] is the heat flow. In other words, the relative current volume corresponds to the area from under the curve between $T_{x}$ and a given temperature to the whole area under the crystallization peak.

**Figure 4:**
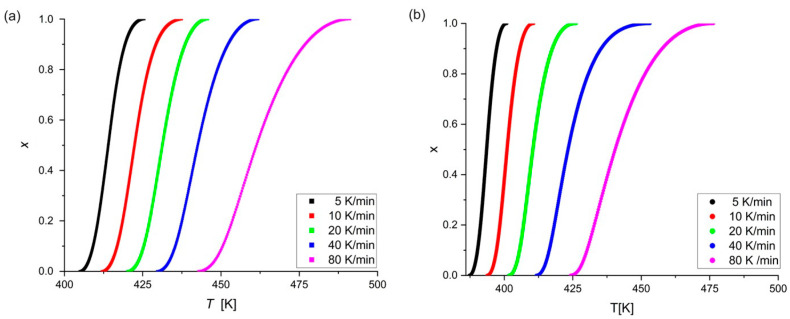
Relative crystallinity of (**a**) Mg_72_Zn_27_Pt_1_, (**b**) first peak of Mg_72_Zn_27_Cu_1_ alloy versus temperature at various heating rates: 5, 10, 20, 40, 80 K/min.

All of the curves in Figure 4 display a characteristic sigmoidal style, as reported for different amorphous alloys during non-isothermal crystallization [3,16,18,20]. The temperature range of the crystallization process is much larger for higher heating rates.

Then, the activation energies of the glass transition, onset crystallization and peak of crystallization were calculated through the six chosen model-free methods based on the determined $T_{g}$, $T_{x}$ and $T_{p}$ values, respectively. The rules of plotting in either approach are collected in Table 2. Every graph shows five points, each of which corresponds to the value of the heating rate. On their basis, a trend line is created, and its slope multiplied by the appropriate parameter (Table 2, column 6) allows for the calculation of the values of the activation energies.

### 3.1. Kissinger Method

**Figure 5:**
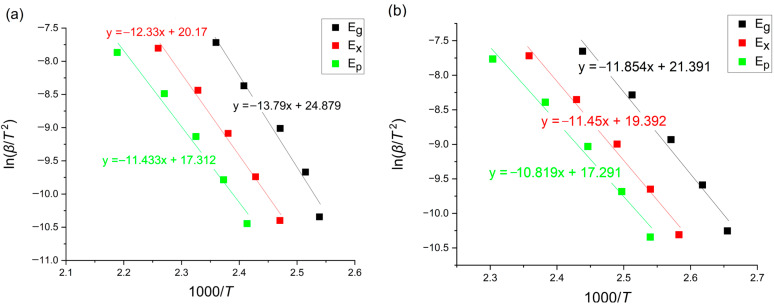
Kissinger plots of amorphous (**a**) Mg_72_Zn_27_Pt_1_ and (**b**) Mg_72_Zn_27_Cu_1_.

According to the popular Kissinger method, the activation energies of the reaction are calculated using the following equation [8,22]:

(2) $$ln\left(\frac{\beta }{T^{2}}\right)=-\frac{E}{RT}+\ln\left(\frac{k_{0}R}{E}\right)$$

where *β* [K/min] is the heating rate, T is a given temperature, *E* [kJ/mol] is an activation energy, *R* is the universal gas constant equaling 8.31 [J/mol K] and $k_{0}[s^{-1}]$ is the pre-exponential factor. The Kissinger plots for the glass transition, onset of crystallization and crystallization peak region are presented in Figure 5.

### 3.2. Ozawa–Flynn–Wall Method (OFW)

**Figure 6:**
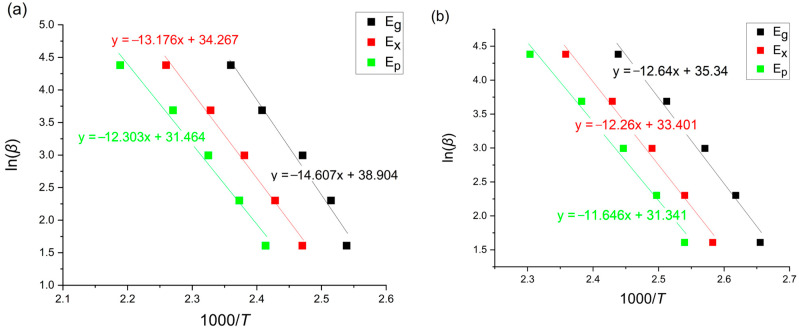
OFW plots of amorphous (**a**) Mg_72_Zn_27_Pt_1_ and (**b**) Mg_72_Zn_27_Cu_1_.

In the OFW method, the activation energies of the reaction are calculated through the following equation [9,10,21]:

(3) $$ln\left(\beta \right)=-1.0516\frac{E}{RT}+const$$

where *β* [K/min] is the heating rate, *E* [kJ/mol] is the activation energy, *R* is the universal gas constant equaling 8.31 [J/molK] and T is a given temperature. The OFW plots for the three analyzed regions are shown in Figure 6.

### 3.3. Boswell Method

**Figure 7:**
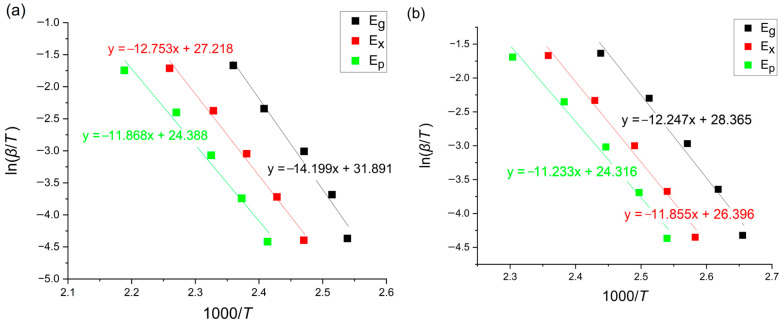
Boswell plots of amorphous (**a**) Mg_72_Zn_27_Pt_1_ and (**b**) Mg_72_Zn_27_Cu_1_.

According to the Boswell method, the values of the activation energies are calculated through the following equation [11,22]:

(4) $$ln\left(\frac{\beta }{T}\right)=-\frac{E}{RT}+const$$

where *β* [K/min] is the heating rate, T is a given temperature, *E* [kJ/mol] is the activation energy and *R* is the universal gas constant equaling 8.31 [J/molK]. The Boswell plots for the glass transition, onset of crystallization and crystallization peak region are presented in Figure 7.

### 3.4. Tang Method

**Figure 8:**
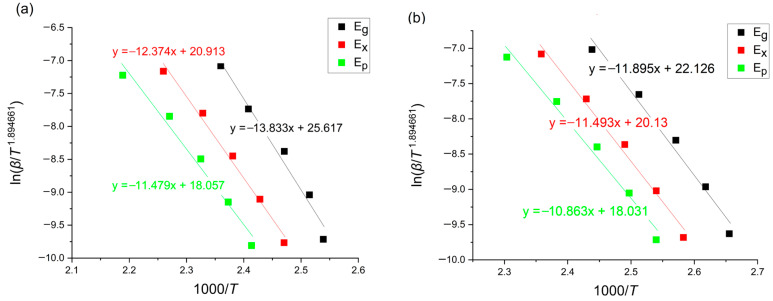
Tang plots of amorphous (**a**) Mg_72_Zn_27_Pt_1_ and (**b**) Mg_72_Zn_27_Cu_1_.

This method is based on the following equation [12,22]:

(5) $$\ln\left(\frac{\beta }{T^{1.894661}}\right)=-1.00145033\frac{E}{RT}+const$$

where *β* [K/min] is the heating rate, T is a given temperature, *E* [kJ/mol] is an activation energy and *R* is the universal gas constant equaling 8.31 [J/molK]. The Tang plots for the glass transition, onset of crystallization and crystallization peak region are presented in Figure 8.

### 3.5. Augis–Bennett Method

**Figure 9:**
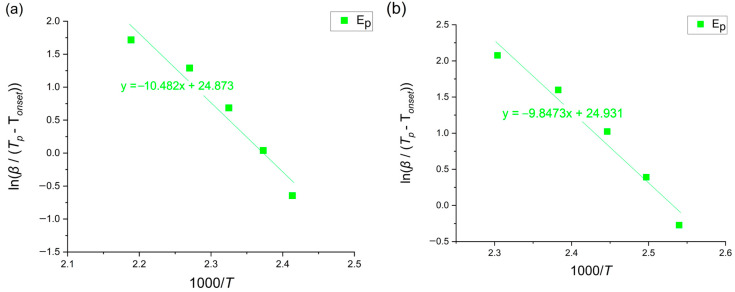
Augis–Bennett plots of amorphous (**a**) Mg_72_Zn_27_Pt_1_ and (**b**) Mg_72_Zn_27_Cu_1_.

This method allows for calculations only after crystallization has started, so, in this case, it is only possible to calculate the value at the peak. The activation energy can be calculated using the following equation [13,22]:

(6) $$ln\left(\frac{\beta }{T-T_{x}}\right)=-\frac{E}{RT}+\ln\left(k_{0}\right)$$

where *β* [K/min] is the heating rate, T [K] is a given temperature, $T_{x}$ [K] is the onset of crystallization temperature, *E* [kJ/mol] is the activation energy, *R* is the universal gas constant equals 8.31 [J/molK] and $k_{0}[s^{-1}]$ is the pre-exponential factor. The Augis–Bennett plots for the crystallization peak region are presented in Figure 9.

### 3.6. Gao–Wang Method

**Figure 10:**
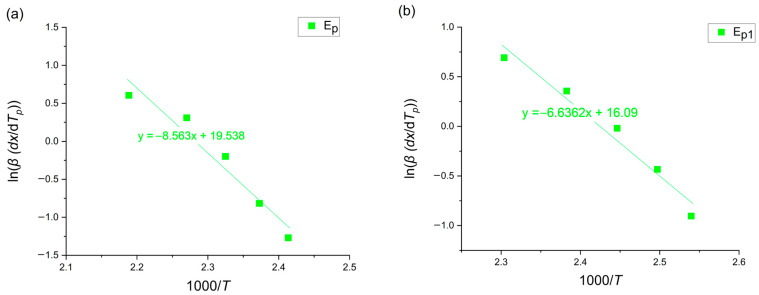
Gao–Wang plot of amorphous (**a**) Mg_72_Zn_27_Pt_1_ and (**b**) Mg_72_Zn_27_Cu_1_.

The Gao–Wang method [14] is a special version of the Friedman method [15], which allows one to also determine the value of the activation energy at the crystallization peak temperature. It includes the dx/dT ratio at the peak point. The activation energy value was calculated through the following equation [14]:

(7) $$\ln{\left(\beta \frac{dx}{dT}\right)}_{p}=-\frac{E}{RT}+const$$

where *β* [K/min] is the heating rate, dx/dT [K] is the increase in the crystallized volume with the given temperature, *E* [kJ/mol] is the activation energy, *R* is the universal gas constant equaling 8.31 [J/molK], T [K] is a given temperature, and the Gao–Wang plot for the crystallization peak region is presented in Figure 10.

All of the calculated activation energies for the transitions are compared in Table 3. The most similar values are obtained using the Kissinger and Tang methods. The OFW method also gives values close to those provided by the Kissinger and Tang methods. The highest calculated values are obtained with the Boswell method (they are also not too different to the others). The Kissinger, Ozawa, Boswell and Tang methods have a similar equation structure and allow for the calculation of the activation energy of all the characteristic regions, i.e., the glass transition, crystallization onset and peak. The Kissinger method is undoubtedly the most common in the literature, which does not necessarily mean it has the highest accuracy. However, methods taking into account additional parameters ($T_{x}$ and dx/dT), such as the Augis–Bennett and Gao–Wang methods, give much lower results. As mentioned, the Augis–Bennett and Gao–Wang methods only allow for the calculation of the activation energy after the start of crystallization, in this case, only for the maximum crystallization peaks $E_{p}$. For the Mg_72_Zn_27_Pt_1_ and the first Mg_72_Zn_27_Cu_1_ peak, the values obtained by the Augis–Bennett method are lower by only about 8 kJ/mol. The lowest and most inconsistent values were obtained using the Gao–Wang method.

The $E_{g},$ $E_{x}\;and\;E_{p}$ values correspond to the energy necessary for atomic rearrangement in the glass transition region, nucleation and crystal growth of each phase, respectively. Under normal conditions, the system tends to lower its energy. For both alloys, $E_{g\;}>E_{x}>E_{p},$ which indicates meta-stability in the amorphous state and that crystallization occurs spontaneously.

The values of the activation energy are higher for Mg_72_Zn_27_Pt_1_, which indicates its greater thermal stability due to the difference in the size of the atoms of all alloy components [16,19]. The atomic radius (van der Waals) equals 173 pm, 139 pm, 209 pm and 140 pm for Mg, Zn, Pt and Cu, respectively [19]. The more diverse the sizes of the constituent atoms in a metallic glass, the more stable it will be.

During non-isothermal annealing, the activation energy is not constant throughout the whole crystallization range. Hence, the values of the local activation energies were also calculated using the six methods described before. In each equation, the *T* parameter is the temperature corresponding to the same degree of crystallized volume (each 0.05) on every DSC curve. The $E_{loc}$ values give information about the average activation energy at each analyzed crystallized volume. Kissinger’s method considering the temperature of the relative volume of the crystallized phase is called the Kissinger–Akahira–Sunose (KAS) modification [22].

**Figure 11:**
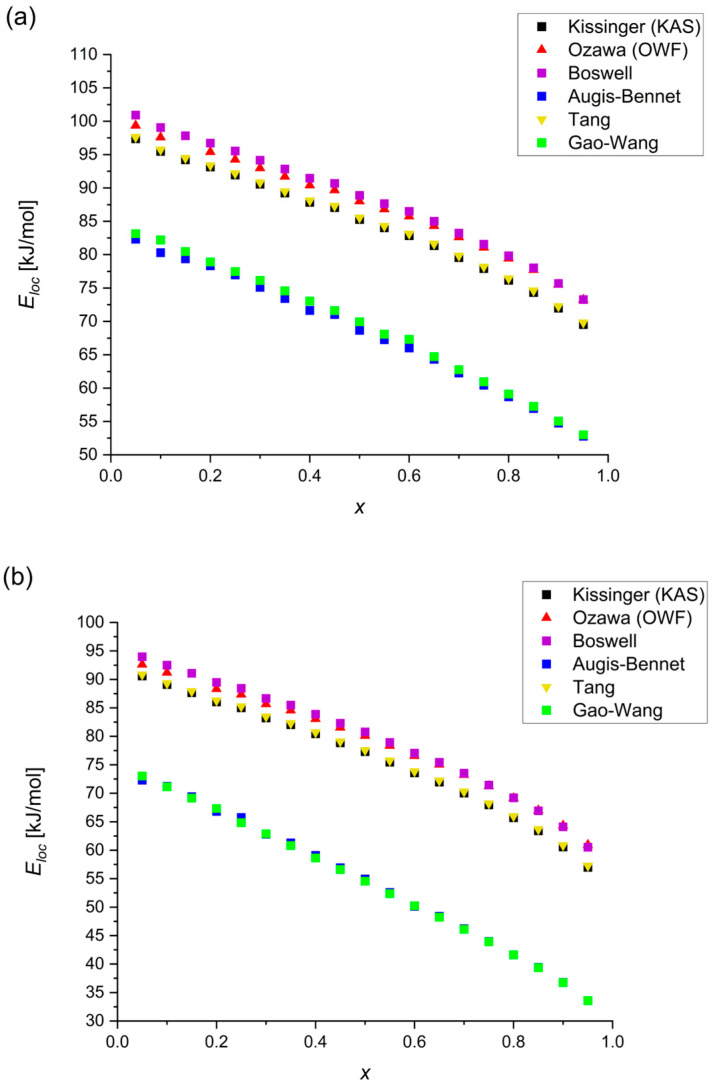
Local values of activation energy for (**a**) Mg_72_Zn_27_Pt_1_; (**b**) first peak of Mg_72_Zn_27_Cu_1_ calculated by various methods.

The values obtained by the Kissinger, Ozawa, Boswell and Tang methods are quite similar. However, the results from the Augis–Bennett and Gao–Wang methods are much lower than those from the others. Despite this, in Figure 11, it is visible that the courses of all of the curves (created from calculated points) are very similar.

Mohammadi Rahvard et al. [23] calculated the values of $E_{g},$ $E_{x1}$, ${\;E}_{p1}$, $E_{x2}$, $E_{p2}$ for the Zr_56_Co_28_Al_16_ and Zr_56_Co_24_Ag_4_Al_16_ alloys using the Kissinger and Ozawa methods. Similarly to this work, these methods gave results relatively close to each other; they, on average, varied from 10 to 15 kJ/mol in each case, while in this work, the differences in the calculations using the Ozawa and Kissinger methods are only of the order of 2–3 kJ/mol. Moreover, as in this work, in [23], each selected method indicates dependencies $E_{x}>E_{p}$ and $E_{p1}>E_{p2}$.

Razei-Shareza et al. [24] analyzed the activation energies of amorphous alloys (Fe_41_Co_7_Cr_15_Mo_14_Y_2_C_15_B_6_)_100-x_Cu_x_ (where *x* = 0, 0.25 and 0.5 at.%) using the Kissinger–Akahira–Sunose (KAS), Ozawa–Flynn–Wall (OFW), Augis–Bennett and Gao–Wang methods. The values of the peak points calculated using these different methods differed significantly in each case. There was no clear relationship between the results, but most often (opposite to the findings of this work), the highest values were obtained using the Gao–Wang or Augis–Bennet methods, and the lowest were found with the OFW method. Additionally, in [24], the local energy values calculated by the KAS and OFW methods were very close, and the curves maintained a similar course.

Zhang [25] in his review describes selected kinetic methods in thermal analysis. Zhang considers the Kissinger and Ozawa methods to be the most popular and intended for simple reactions. According to [25], the Boswell and Augis–Bennett methods are methods intended for transformations in the solid state, whereas the Friedmann method (and also the Gao–Wang method based on the Friedmann method) shows a greater accuracy for complex systems. Therefore, methods based on Friedmann and intended for the analysis of transformations in a solid may provide greater accuracy for investigating alloys.

For analyzing complex crystallization processes with random nucleation, Zhang [25] recommends the Kissinger–Akahira–Sunose (KAS) and Matusita–Sakka modifications. The KAS method was used in this work; however, it does not differ in accuracy from other methods that take into account the dependence of temperature of the relative crystallized volume, such as the OWF and Tang local methods. In contrast, the Matusita–Sakka method includes additional parameters like the Avrami exponent *n* and the coefficient corresponding to the dimensionality of crystal growth *m*. Therefore, it is no longer a simple model.

A similar analysis of the local transformation activation energy was used for the non-isothermal annealing of LiFePO_4_ olivine using the KAS and OFW methods [26]. The values obtained using these two methods were also close to each other; however, higher values (about 10–15 kJ/mol) were obtained with the Kissinger method, unlike the results from this publication. The authors will use the results from the Kissinger method in further analyses.

## 4. Conclusions

Based on the research conducted, the following conclusions can be drawn:

1. Crystallization proceeds with one exothermic peak for amorphous Mg_72_Zn_27_Pt_1_ alloy and with two exothermic peaks for amorphous Mg_72_Zn_27_Cu_1_ alloy, respectively.
2. The characteristic temperatures ${\;T}_{g}$, $T_{x}$ and $T_{p}$ are strongly dependent on the heating rate during non-isothermal annealing. The addition of Pt and Cu increases the characteristic temperatures toward higher values and, consequently, the stability of metallic glass compared to two-component Mg_72_Zn_28_ glass [17], especially that with Pt, due to its higher melting point and different atom size to those of Mg and Zn.
3. The activation energies for the amorphous Mg_72_Zn_27_Pt_1_ alloy fluctuate in the range of 114.60–117.99 kJ/mol, 102.46–105.98 kJ/mol and 71.16–98.62 kJ/mol for $E_{g}$, $E_{x}$ and $E_{p}$, respectively, whereas, for Mg_72_Zn_27_Cu_1_, the calculated values are in the range of 98.51–101.77 kJ/mol, 95.15–98.51 kJ/mol and 55.15–93.34 kJ/mol for $E_{g}$, $E_{x}$ and $E_{p}$, respectively.
4. Based on the $E_{g},$ $E_{x}$, ${\;E}_{p}$ and local energy values, it can be confirmed that both alloys are meta-stable in the amorphous state and crystallization occurs spontaneously.
5. The most similar values of the activation energy for three characteristic regions are given by the Kissinger, Ozawa, Tang and Boswell methods, because the structures of their equations are similar. The Boswell method gives the highest results, and the Augis–Bennett and Gao–Wang methods give significantly lower results.
6. The Augis–Bennett and Gao–Wang methods allow for the calculation of the activation energy at the crystallization peak and they are the only ones that consider $T_{x}$ or dx/dT.
7. The use of the Gao–Wang method to analyze an alloy with two crystallization peaks may be difficult due to the inclusion of dx/dT.
8. The Augis–Bennett method, despite only slightly lower values for the peak activation energies, shows significantly lower values for the local activation energy.
9. Taking into account the ease of their formulas, their best convergence, and their widespread use in the literature, the KAS and OFW methods will work very well for all comparisons.

## Figures and Tables

**Table 1:** Values of characteristic temperatures for Mg_72_Zn_27_Pt_1_ and Mg_72_Zn_27_Cu_1_ alloy at various heating rates.

Alloy	*β* [K/min]	$T_{g}$ [K]	$T_{x}$ [K]	$T_{p}$ [K]	$T_{x\_end}$ [K]	$T_{x2}$ [K]	$T_{p2}$ [K]	$T_{x2\_end}$ [K]
Mg_72_Zn_27_Pt_1_	5	393.90	404.82	414.35	426.88	—	—	—
	10	397.69	411.85	421.45	437.47	—	—	—
	20	404.75	420.07	430.14	448.85	—	—	—
	40	415.29	429.50	440.50	461.86	—	—	—
	80	423.80	442.57	456.93	491.17	—	—	—
Mg_72_Zn_27_Cu_1_	5	376.64	387.20	393.76	400.90	487.92	502.72	510.39
	10	382.00	393.72	400.48	410.69	505.90	519.56	529.39
	20	389.00	401.60	408.79	426.25	528.08	540.29	557.17
	40	398.00	411.63	419.71	453.18	551.02	565.14	588.27
	80	410.11	424.06	434.08	476.35	579.91	591.59	617.22

**Table 2:** The rules of plotting and the parameters for slope multiplying for each method.

Method	Glass Transition	Onset of Crystallization	Crystallization Peak	Parameter for Slope Multiplying
Kissinger	$ln\left(\frac{\beta }{{T_{g}}^{2}}\right)$ $\;\mathrm{vs}.\;\frac{1000}{T_{g}}$	$ln\left(\frac{\beta }{{T_{x}}^{2}}\right)$ $\;\mathrm{vs}.\;\frac{1000}{T_{x}}$	$ln\left(\frac{\beta }{{T_{p}}^{2}}\right)$ $\;\mathrm{vs}.\;\frac{1000}{T_{p}}$	(−R)
Ozawa–Flynn–Wall	$ln\left(\beta \right)$ $\;\mathrm{vs}.\;\frac{1000}{T_{g}}$	$ln\left(\beta \right)$ $\;\mathrm{vs}.\;\frac{1000}{T_{x}}$	$ln\left(\beta \right)$ $\;\mathrm{vs}.\;\frac{1000}{T_{p}}$	$\left(-\frac{R}{1.0516}\right)$
Boswell	$ln\left(\frac{\beta }{T_{g}}\right)$ $\;\mathrm{vs}.\;\frac{1000}{T_{g}}$	$ln\left(\frac{\beta }{T_{x}}\right)$ $\;\mathrm{vs}.\;\frac{1000}{T_{x}}$	$ln\left(\frac{\beta }{T_{p}}\right)$ $\;\mathrm{vs}.\;\frac{1000}{T_{p}}$	(−R)
Tang	$ln\left(\frac{\beta }{{T_{g}}^{1.894661}}\right)$ $\;\mathrm{vs}.\;\frac{1000}{T_{g}}$	$ln\left(\frac{\beta }{{T_{x}}^{1.894661}}\right)$ $\;\mathrm{vs}.\;\frac{1000}{T_{x}}$	$ln\left(\frac{\beta }{{T_{p}}^{1.894661}}\right)$ $\;\mathrm{vs}.\;\frac{1000}{T_{p}}$	$\left(-\frac{R}{1.00145033}\right)$
Augis–Bennett	−	−	$ln\left(\frac{\beta }{T_{p}-T_{x}}\right)$ $\;\mathrm{vs}.\;\frac{1000}{T_{p}}$	(−R)
Gao–Wang	−	−	$\ln\left(\beta \frac{dx}{dT_{p}}\right)$ $\;\mathrm{vs}.\;\frac{1000}{T_{p}}$	(−R)

**Table 3:** Values of activation energies of Mg_72_Zn_27_Pt_1_ and Mg_72_Zn_27_Cu_1_ calculated by various methods.

Alloy	Method	$E_{g}$ [kJ/mol]	$E_{x}$ [kJ/mol]	$E_{p}$ [kJ/mol]
Mg_72_Zn_27_Pt_1_	Kissinger	114.60	102.46	95.01
	Ozawa–Flynn–Wall	115.43	104.12	97.22
	Boswell	117.99	105.98	98.62
	Tang	114.79	102.68	95.25
	Augis–Bennett	−	−	87.11
	Gao–Wang	−	−	71.16
Mg_72_Zn_27_Cu_1_	Kissinger	98.51	95.15	89.91
	Ozawa–Flynn–Wall	99.88	96.88	92.03
	Boswell	101.77	98.51	93.34
	Tang	98.71	95.37	90.14
	Augis–Bennett	−	−	81.83
	Gao–Wang	−	−	55.15

## Data Availability

The data presented in this study are available on request from the authors—A.P. pierwola@agh.edu.pl.
